# Supplementary material for: Molecular engineering improves antigen quality and enables integrated manufacturing of a trivalent subunit vaccine candidate for rotavirus
Source: Microb Cell Fact. 2021 May 1;20:94. doi: 10.1186/s12934-021-01583-6 (PMC8088319; doi:10.1186/s12934-021-01583-6)
Supplement: Supplementary file 1 — Additional file 1. Additional Tables and Figures. [file 12934_2021_1583_MOESM1_ESM.docx]

**Molecular engineering improves antigen quality and enables integrated manufacturing of a trivalent subunit vaccine candidate for rotavirus**

**Supplemental Information**

Neil C. Dalvie^1,2^^ and Joseph R. Brady^1,2^^, Laura E. Crowell^1,2^, Mary Kate Tracey^2^, Andrew M. Biedermann^1,2^, Kawaljit Kaur^3^, John M. Hickey^3^, D. Lee Kristensen II^2^, Alexandra Bonnyman^1,2^, Sergio A. Rodriguez-Aponte^2,4^, Charles A. Whittaker^2^, Marina Bok^5^, Celina Vega^5^, Tarit Mukhopadhyay^6^, Sangeeta B. Joshi^3^, David B. Volkin^3^, Viviana Parreño^5^, Kerry R. Love^1,2^, J. Christopher Love^1,2^*

^1^Department of Chemical Engineering, Massachusetts Institute of Technology, Cambridge, Massachusetts 02139, United States

^2^The Koch Institute for Integrative Cancer Research, Massachusetts Institute of Technology, Cambridge, Massachusetts 02139, United States

^3^Department of Pharmaceutical Chemistry, Vaccine Analytics and Formulation Center, University of Kansas, Lawrence, Kansas, 66047, United States

^4^Department of Biological Engineering, Massachusetts Institute of Technology, Cambridge, Massachusetts 02139, United States

^5^Instituto de Virología e Innovaciones Tecnológicas, IVIT, CONICET-INTA,, Hurlingham, Buenos Aires, Argentina

^6^Department of Biochemical Engineering, University College London, Gower Street, London, WC1E 6BT, United Kingdom

^Contributed equally

*Correspondence to: [clove@mit.edu](mailto:clove@mit.edu)

**A**


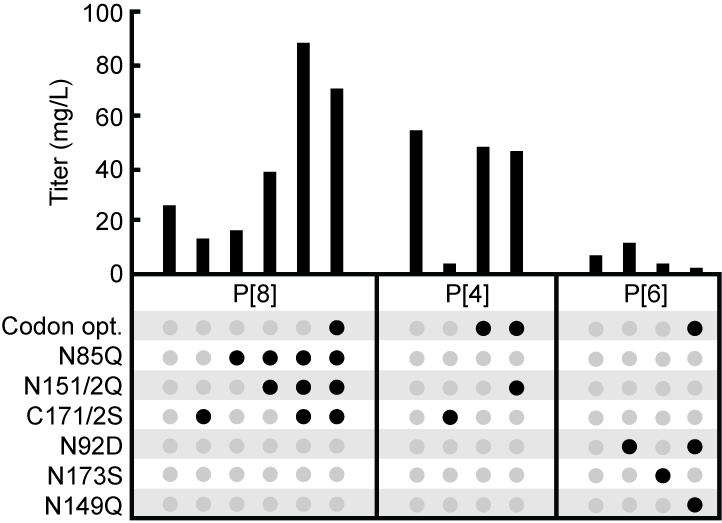


**B**


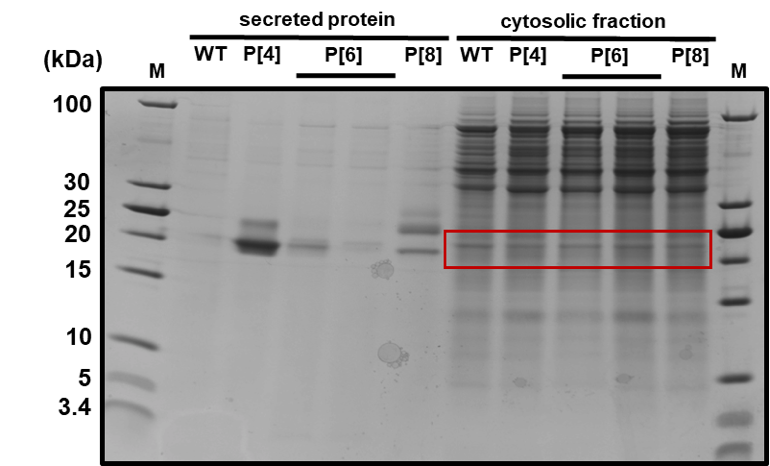


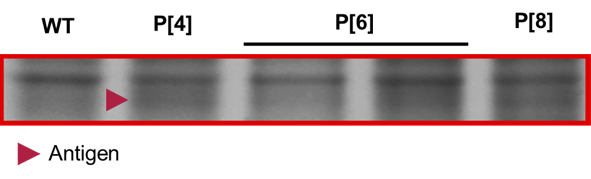


**Fig. S1. Titers of antigen expression.** A) Secreted titers of each NRRV antigen variant produced in 3 mL, plate-based cultures. Titer was calculated using SDS-PAGE and densitometry, with comparison to a known standard. B) SDS-PAGE of supernatant and intracellular lysates from expression of original serotypes. No significant amount of P[6] antigen was detected in cell lysates.

**A**


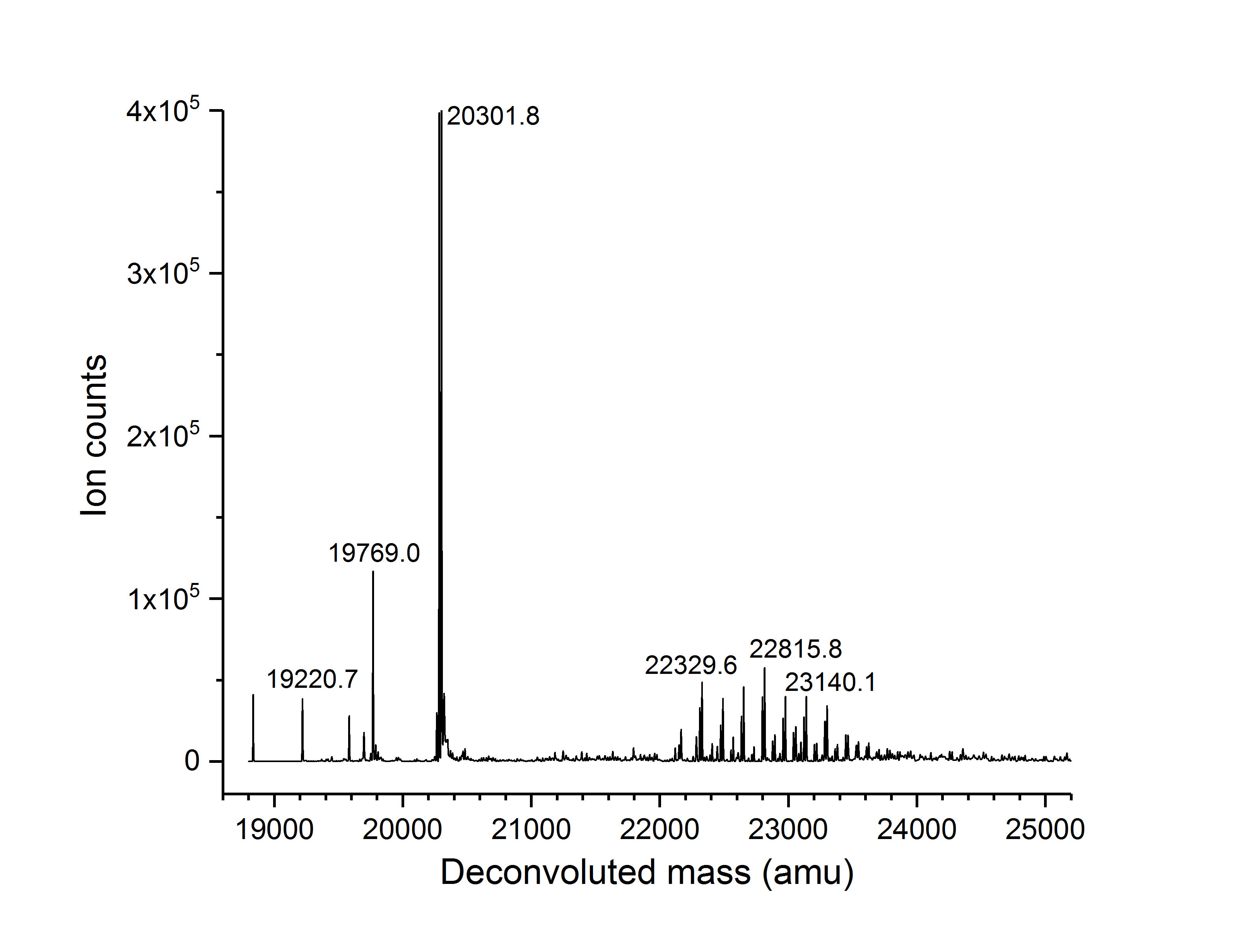


**B**


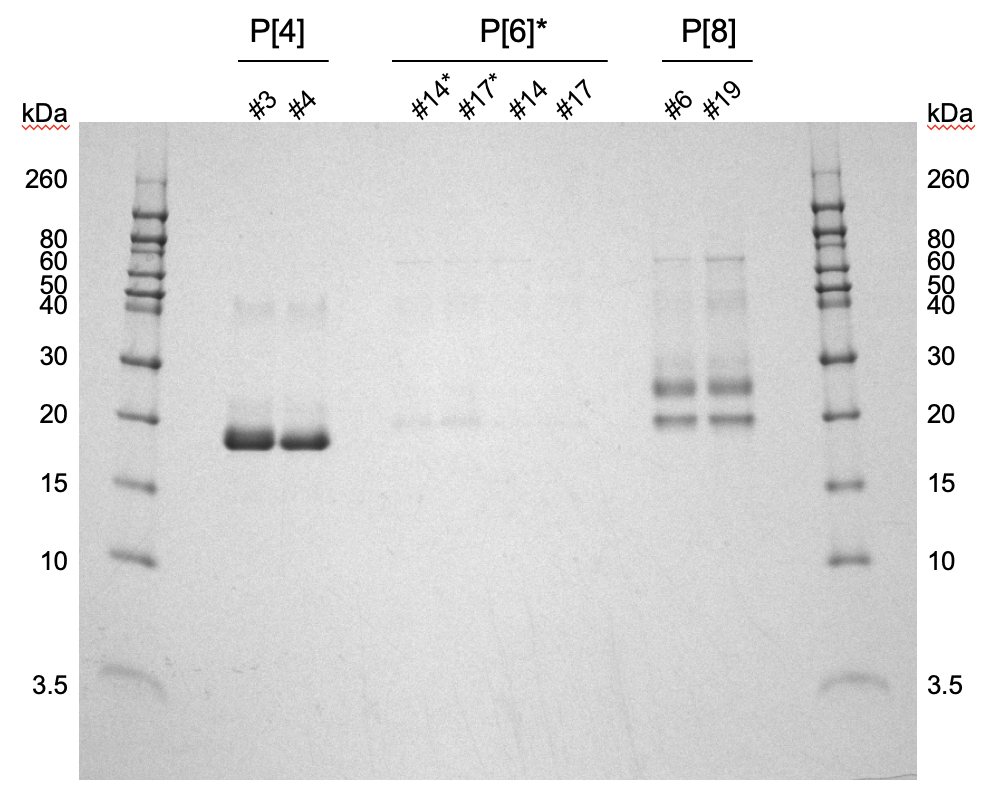


**Fig. S2. Quality analyses of antigens.** A) Mass spectrum of P[8] from intact LCMS showing putative full-length, aglycosylated P[8] (~20.3 kDa) and high-mannose variants of P[8] (>22 kDa). B) Non-reduced SDS-PAGE of P[4], P[6], and P[8] supernatants. P[6] samples with an asterisk are concentrated 5x. Dimer and higher aggregates are visible in P[4] and P[8] lanes.

**A**


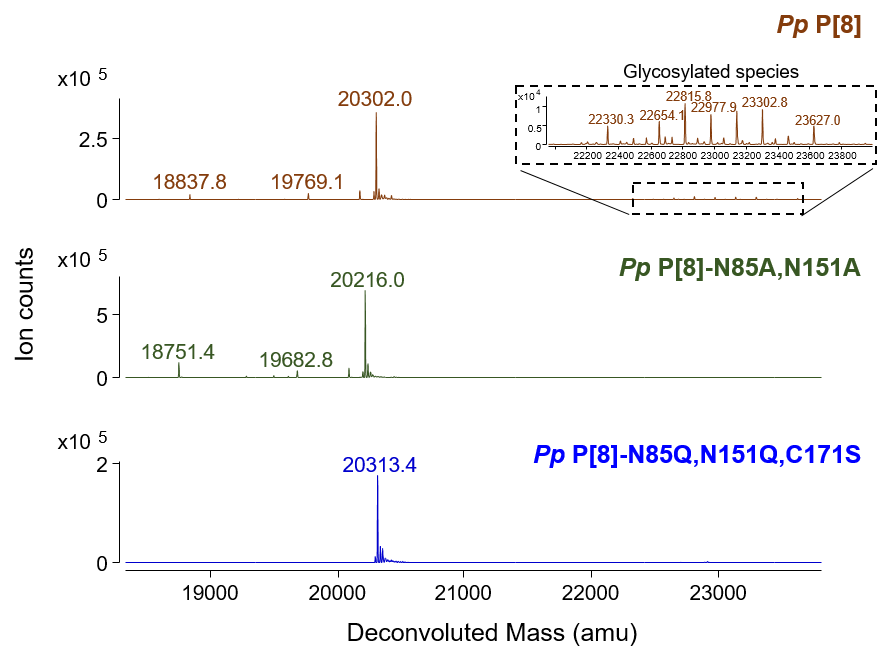


**Fig. S3. Glycosylation removal in P[8].** A) Mass spectra of P[8] variants, indicating the removal of hypermannosylated product variants with the introduction of N85Q and N151Q mutations.


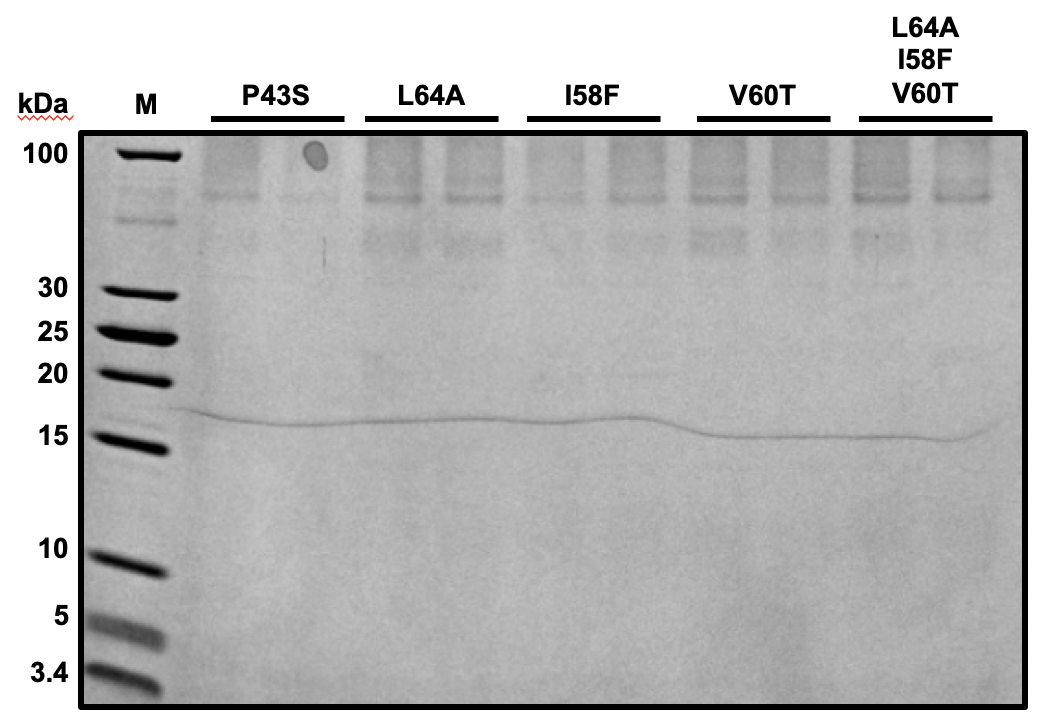


**Fig. S4. Hydrophobic region mutations in P[6].** A) SDS-PAGE of sequence variants of P[6] with reduced hydrophobicity. Expected product size is ~20 kD.


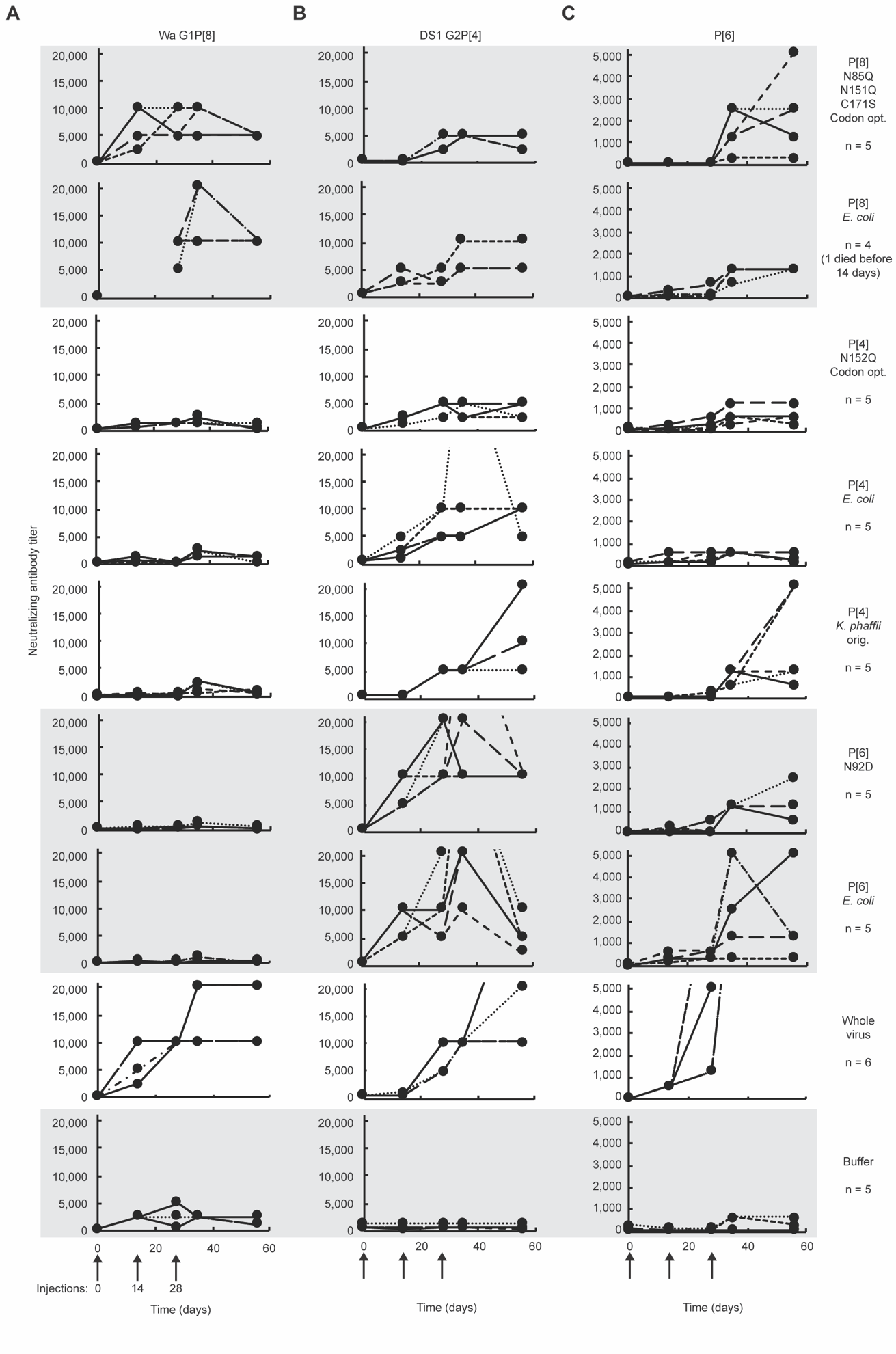


**D**


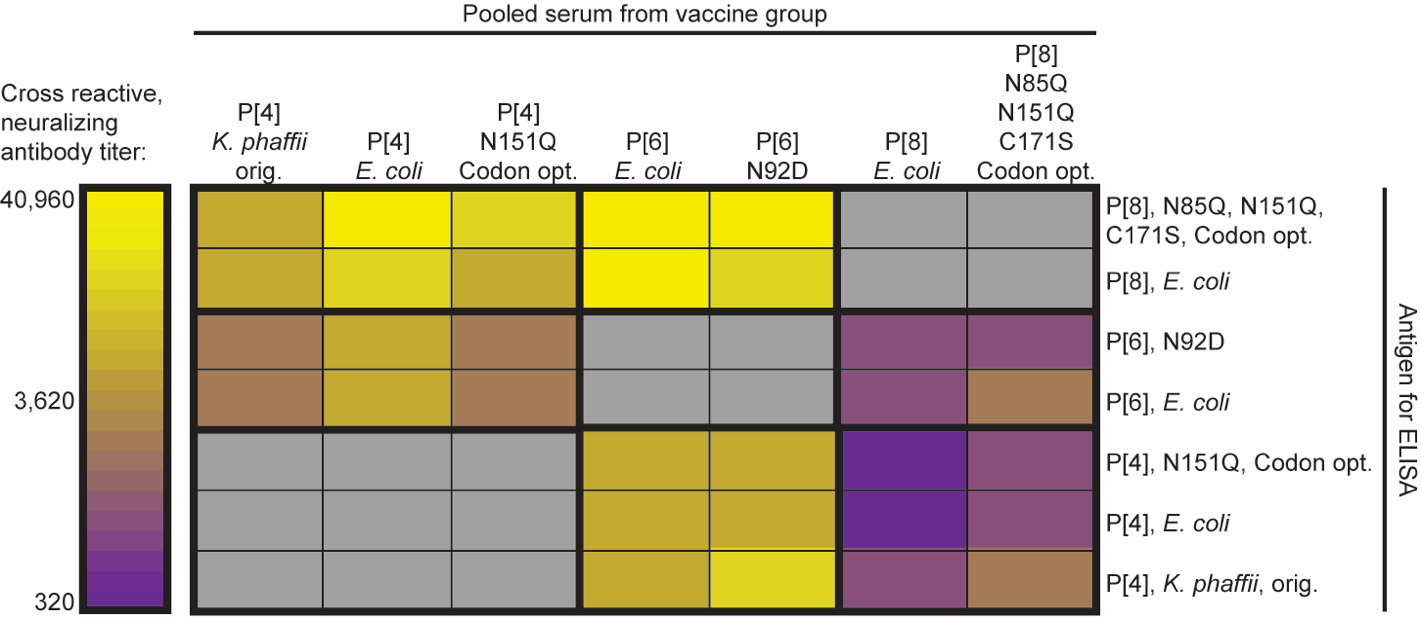


**Fig. S5. Animal study results.** Titer over time of neutralizing antibodies generated in guinea pigs against A) P[8], B) P[4], and C) P[6] virus strains. Each line represents one animal, vaccinated with the antigen indicated at right. Animals were immunized at 0, 14, and 28 days for a total of three doses. Lines extending above the chart limits indicate a titer of 40,960, or 10,240 for ST3 G4P[6]. D) Cross reactivity of serum across all seven antigens by ELISA for IgG antibodies. Serum from all animals in each group was pooled.


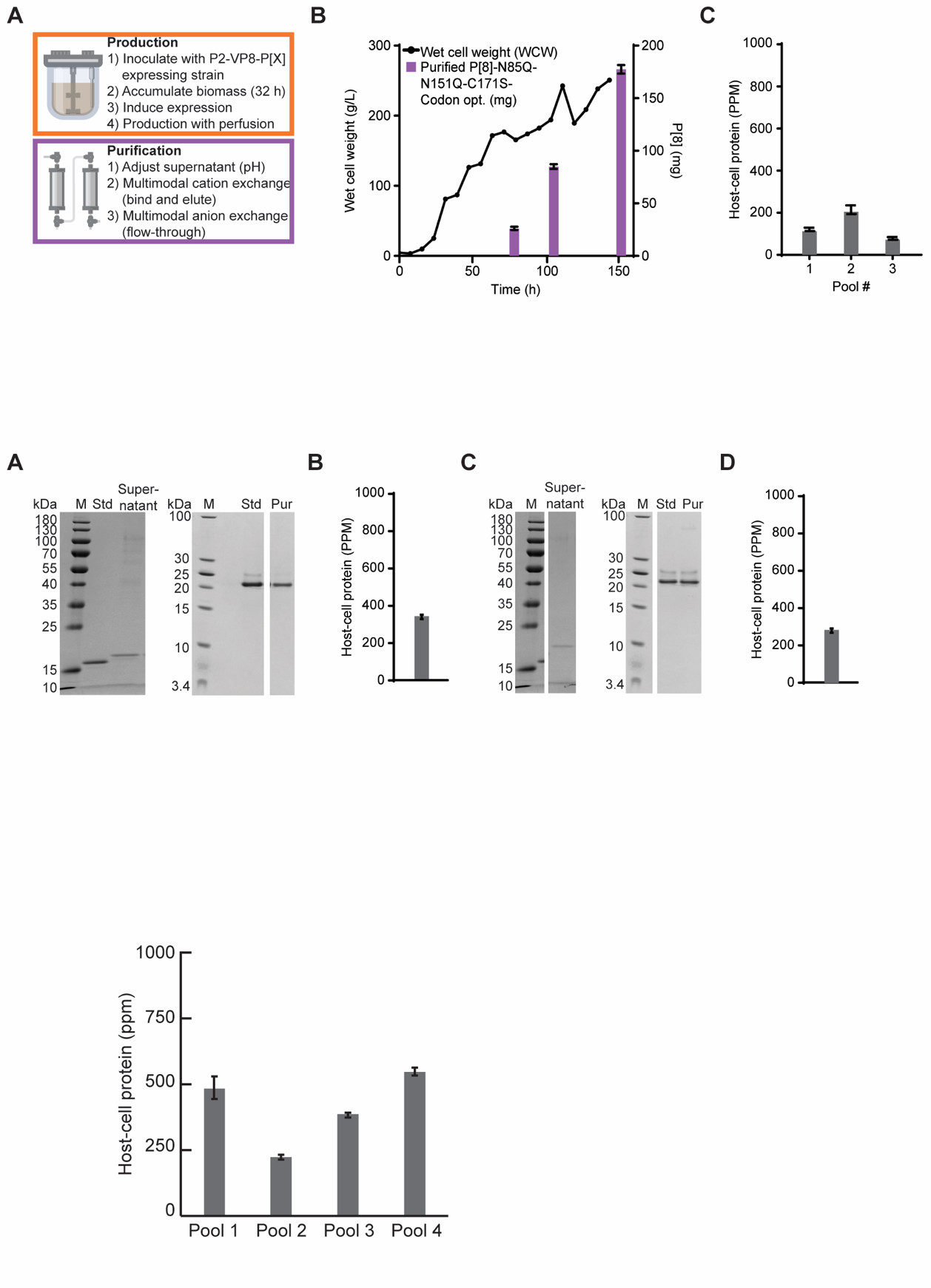


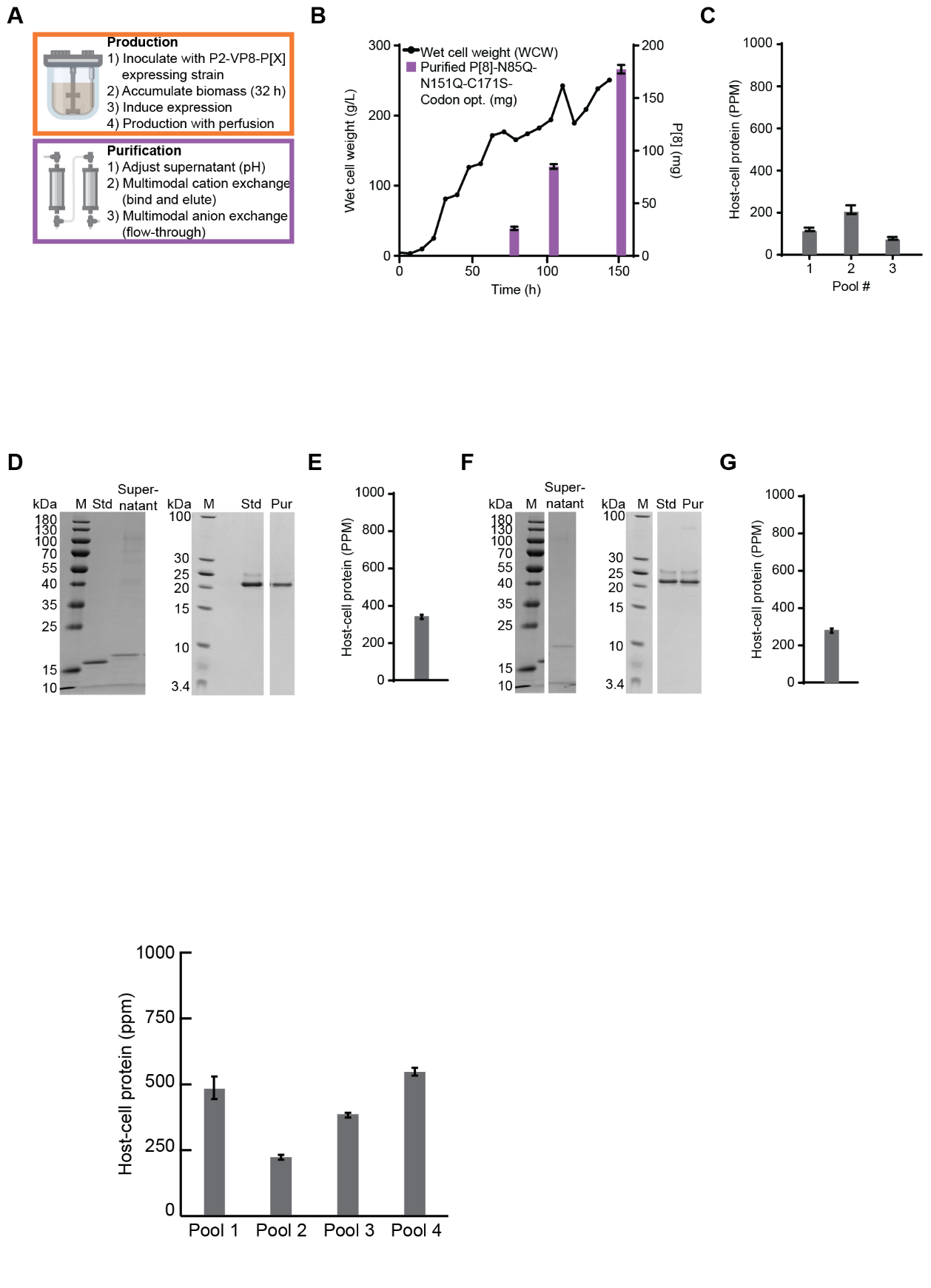


**Fig. S6. Process development of individual antigens.** A) Schematic and B,C) process development data for an end-to-end production run of engineered P[8]. D-G) Purification development of engineered versions of P[4] (D,E), and P[6] (F,G). Cells were cultured in shake flasks, and supernatant was purified using the same process as engineered P[8].

**A B**


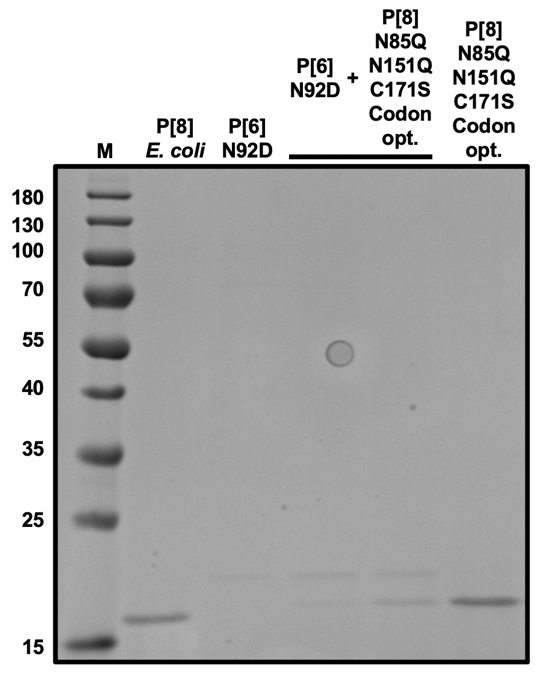

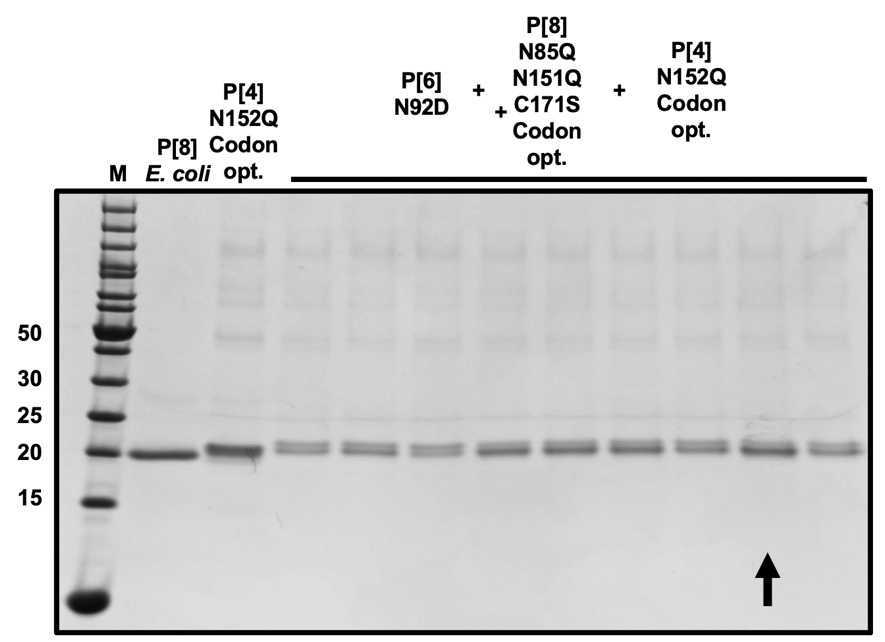


**C D**


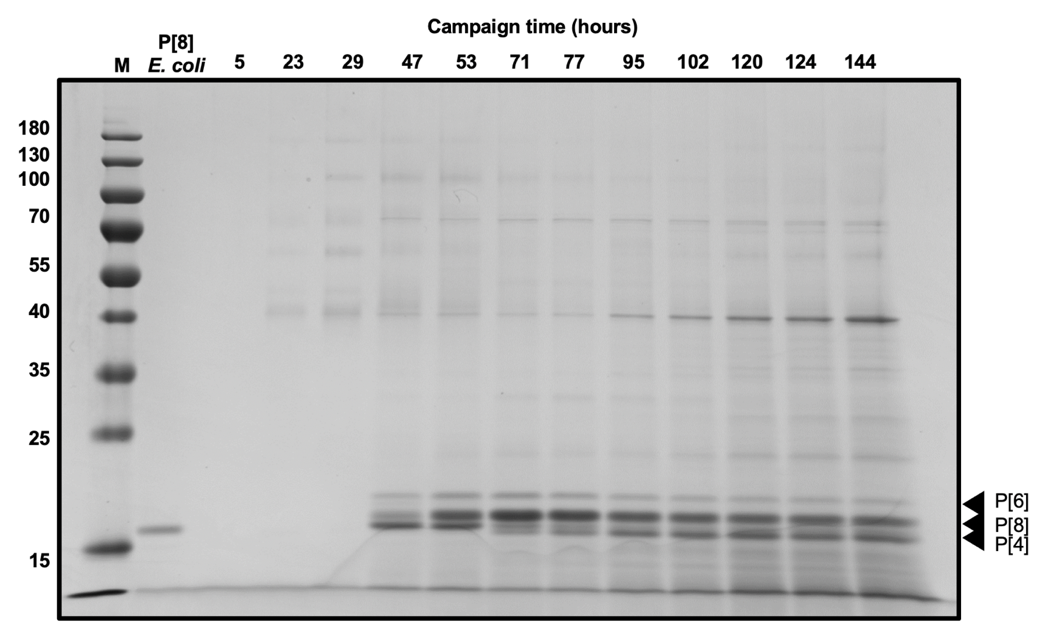
 **
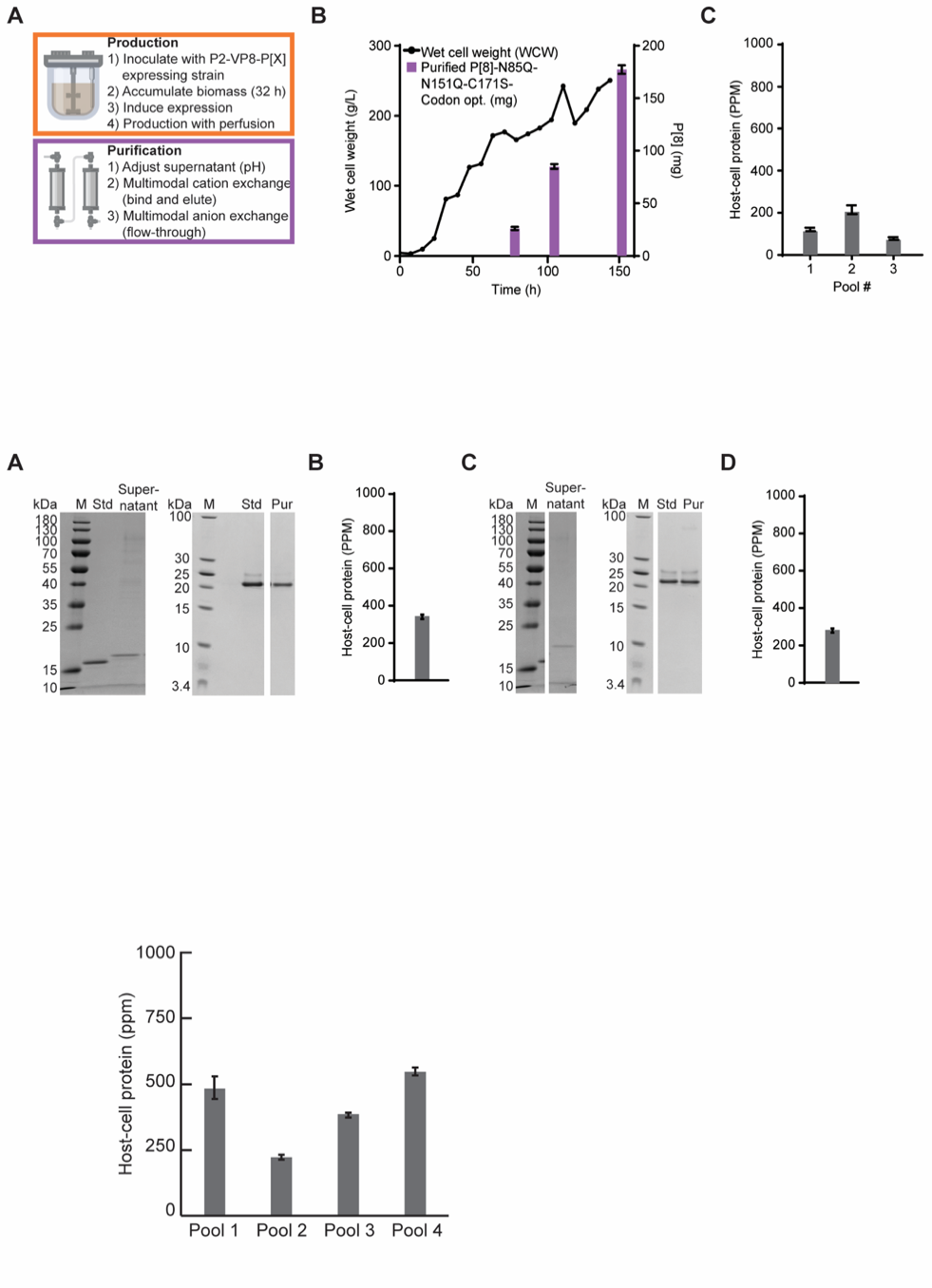
**

**Fig. S7. Expression of all three NRRV antigens.** A) SDS-PAGE of supernatant of two clones that express engineered P[8] in a background strain expressing engineered P[6]. B) SDS-PAGE of supernatant of cells that express all three antigens. Cells were cultivated at 3 mL plate scale. The strain marked with an arrow was carried forward to reactor scale. C) SDS-PAGE of bioreactor samples across the campaign. All three engineered antigens are visible by SDS-PAGE. D) Concentration of host cell protein in purified product pools, as measured by host cell protein ELISA for *K. phaffii*.

Table S1. Accessibility scores of potential *N*-linked glycosylation sites in P[8], identified with NetNGlyc.

| Index | N-X-S/T | Accessibility score |
| --- | --- | --- |
| 53 | N-N-S | 0.2 |
| 85 | N-V-S | 0.7 |
| 88 | N-D-S | 0.2 |
| 151 | N-I-S | 0.9 |

Table S2. Normalized enrichment scores for gene sets significantly enriched (p-adj <0.05) in genes differentially expressed between serotype-expressing strains.

| **Gene ontology - biological process** | **P[6] vs. Null** | **P[6] vs. P[4]** | **P[6] vs. P[8]** |
| --- | --- | --- | --- |
| cytoplasmic translation | 2.19 | 2.52 | 2.19 |
| translocation | 1.92 | 1.81 |  |
| protein glycosylation | 2.13 | 2.22 | 2.02 |
| cell wall organization or biogenesis | 2.38 | 2.18 | 2.28 |
| DNA replication | 1.78 | 1.96 | 2.20 |
| carbohydrate metabolic process | 1.68 | 1.88 | 2.18 |
| DNA repair |  | 1.79 | 2.16 |
| telomere organization | 1.89 | 1.78 | 1.95 |
| cell budding | 1.80 | 1.72 | 1.97 |
| DNA recombination |  | 1.66 | 2.04 |
| cellular response to DNA damage stimulus |  | 1.63 | 2.02 |
| signaling | 1.73 | 1.55 | 1.65 |
| cytoskeleton organization | 1.68 | 1.51 | 2.11 |
| Golgi vesicle transport | 1.65 | 1.51 | 1.39 |
| cell morphogenesis |  |  | 1.84 |
| chromatin organization |  |  | 1.50 |
| chromosome segregation |  |  | 1.97 |
| cofactor metabolic process |  |  | 1.63 |
| conjugation | 1.78 |  | 1.80 |
| cytokinesis |  |  | 1.75 |
| generation of precursor metabolites | -1.74 |  | 1.82 |
| meiotic cell cycle |  |  | 1.89 |
| mitochondrial translation |  |  | -2.01 |
| mitotic cell cycle | 1.62 |  | 1.93 |
| monocarboxylic acid metabolic process |  |  | 1.61 |
| mRNA processing |  |  | -1.61 |
| small molecule metabolic process |  |  | 2.06 |
| organelle fission |  |  | 1.96 |
| protein folding |  |  | -2.26 |
| regulation of cell cycle |  |  | 1.70 |
| regulation of protein modification |  |  | 1.57 |
| regulation of transport | 1.68 |  |  |
| ribosomal large subunit biogenesis |  |  | -1.52 |
| ribosomal small subunit biogenesis | 1.81 |  | -1.72 |
| RNA modification |  |  | -2.12 |
| RNA splicing |  |  | -1.63 |
| rRNA processing | 1.70 |  | -2.10 |
| sporulation |  |  | 1.59 |
| transposition |  |  | 1.69 |
| tRNA processing |  |  | -1.77 |
| vitamin metabolic process | -2.41 |  |  |

Table S3. Animal study groups.

| Host | Antigen | N | Notes |
| --- | --- | --- | --- |
| E. coli | P[8] | 5 | 1 animal died |
| E. coli | P[6] | 5 |  |
| E. coli | P[4] | 5 |  |
| K. phaffii | P[8]-N85Q-N151Q-C171S-Codon opt. | 5 |  |
| K. phaffii | P[6]-N92D | 5 |  |
| K. phaffii | P[4]-N152Q-Codon opt. | 5 |  |
| K. phaffii | P[4] | 5 |  |
| --- | RVA trivalent | 6 |  |
| --- | Buffer | 5 |  |

Table S4. DNA seqences of optimized NRRV antigens.

| Antigen | DNA sequence |
| --- | --- |
| P[8]_N85Q_N151Q_C171S_CodonOpt | CAGTACATCAAGGCTAACTCTAAATTTATTGGTATAACTGAACTAGGATCAGGATCAGGTTTAGACGGACCATATCAACCAACAACTTTCACTCCACCAAACGACTACTGGATCCTGATCAACTCCAACACCAACGGTGTTGTTTACGAGTCCACTAACAACTCCGATTTCTGGACTGCTGTTGTTGCTATCGAGCCACACGTTAACCCAGTTGACAGACAGTACACTATCTTCGGTGAGTCCAAGCAGTTCcaaGTTTCCAACGACTCCAACAAGTGGAAGTTCTTGGAGATGTTCAGATCCTCCAGCCAGAACGAGTTCTACAACAGAAGAACTTTGACCTCCGACACCAGATTGGTCGGTATCTTGAAGTACGGTGGTAGAGTTTGGACTTTCCACGGTGAAACTCCAAGAGCTACTACTGACTCTTCCTCCACTGCCAACTTGAACcaaATTTCCATCACCATCCACTCCGAATTCTACATCATCCCAAGATCTCAAGAGTCCAAGTctAACGAGTACATTAACAACGGTCTG |
| P[4]_N152Q_CodonOpt | CAGTACATCAAGGCTAACTCTAAATTTATTGGTATAACTGAACTAGGATCAGGATCAGGTGTCTTAGACGGACCATATCAACCAACAACTTTCAAGCCACCAAACGACTACTGGTTGCTGATCTCCTCTAACACCAACGGTGTTGTTTACGAGTCCACCAACAACAACGATTTCTGGACTGCTGTTATCGCCGTTGAACCACACGTTTCCCAAACTAACAGACAGTACATCCTGTTCGGTGAGAACAAGCAGTTCAACGTCGAAAACAACTCCGACAAGTGGAAGTTCTTCGAGATGTTCAAGGGTTCTTCCCAGGGTGACTTCTCCAACAGAAGAACTTTGACCTCCTCCAACAGGTTGGTCGGTATGTTGAAGTACGGTGGTAGAGTTTGGACTTTCCACGGTGAAACTCCAAGAGCTACTACCGACTCTTCTAACACTGCCGACTTGAACcaaATCTCCATCATCATCCACTCCGAGTTCTACATCATCCCAAGATCTCAAGAGTCCAAGTGCAACGAGTACATTAACAACGGTCTG |
| P[6]_N92D | CAGTACATCAAGGCCAACTCCAAGTTCATCGGTATCACTGAGCTTGGATCTGGTTCCGGTGTTTTGGATGGTCCTTACCAGCCAACTTCTTTCAAGCCACCAAACGACTACTGGATCTTGCTGAACCCAACTAACCAGCAGGTTGTCTTGGAGGGAACTAACAAGACTGACATCTGGGTCGCCTTGTTGTTGGTTGAACCTAACGTTACCAACCAGTCCAGACAGTACACTCTGTTCGGTGAGACTAAGCAGATCACCGTTGAGAACAACACCgACAAGTGGAAGTTCTTCGAGATGTTCAGAAGGTCCGTTTCCGCTGAGTTCCAGCACAAGAGAACTTTGACTTCCGACACTAAGCTGGCCGGTTTCTTGAAGTTCTACAACTCCGTTTGGACCTTCTACGGTGAAACTCCACACGCTACTACTGACTACTCTTCCACTTCTAACTTGTCCGAGGTTGAGACTGCTATCCACGTCGAGTTCTACATCATCCCAAGATCTCAAGAGTCCAAGTGCAACGAGTACATTAACACCGGTCTG |
